# Characterization of *PISTILLATA*-like Genes and Their Promoters from the Distyly *Fagopyrum esculentum*

**DOI:** 10.3390/plants11081047

**Published:** 2022-04-12

**Authors:** Wei You, Xiangjian Chen, Lingtian Zeng, Zhiyuan Ma, Zhixiong Liu

**Affiliations:** College of Horticulture and Gardening, Yangtze University, Jingzhou 434025, China; 201971435@yangtzeu.edu.cn (W.Y.); 202071696@yangtzeu.edu.cn (X.C.); 202072800@yangtzeu.edu.cn (L.Z.); 202071704@yangtzeu.edu.cn (Z.M.)

**Keywords:** buckwheat, floral development, floral homeotic gene, MADS-box gene, stamen development

## Abstract

*Arabidopsis* *PISTILLATA* (*PI*) encodes B-class MADS-box transcription factor (TF), and works together with *APETALA3* (*AP3*) to specify petal and stamen identity. However, a small-scale gene duplication event of *PI* ortholog was observed in common buckwheat and resulted in *FaesPI_1* and *FaesPI_2*. *FaesPI_1* and *FaesPI_2* were expressed only in the stamen of dimorphic flower (thrum and pin) of *Fagopyrum esculentum*. Moreover, intense beta-glucuronidase (GUS) staining was found in the entire stamen (filament and anther) in *pFaesPI_1::GUS* transgenic *Arabidopsis*, while *GUS* was expressed only in the filament of *pFaesPI_2::GUS* transgenic *Arabidopsis.* In addition, phenotype complementation analysis suggested that *pFaesPI_1::FaesPI_1*/*pFaesPI_2::FaesPI_2* transgenic *pi-1 Arabidopsis* showed similar a flower structure with stamen-like organs or filament-like organs in the third whorl. This suggested that *FaesPI_2* only specified filament development, but *FaesPI_1* specified stamen development. Meanwhile, *FaesPI_1* and *FaesPI_2* were shown to function redundantly in regulating filament development, and both genes work together to require a proper stamen identity. The data also provide a clue to understanding the roles of *PI-like* genes involved in floral organ development during the early evolution of core eudicots and also suggested that *FaesPI_1* and *FaesPI_2* hold the potential application in bioengineering to develop a common buckwheat male sterile line.

## 1. Introduction

Common buckwheat (*Fagopyrum esculentum*) grains are gluten-free and with low-calories, but are rich in bioactive compounds (such as rutin, quercetin, polysaccharides, etc.) [1]. Hence, common buckwheat grains have increased demand for a great potential functional food with illness prevention and health benefits in recent years. However, common buckwheat is a heteromorphic self-incompatibility (SI) crop due to its distylous flowers (pin and thrum), with populations being equally composed of pin and thrum plants [2,3]. In pin plants, long styles are combined with short stamens and small pollen grains; in thrum plants, short styles are combined with long stamens and large pollen grains. Moreover, legitimate cross-pollinations occur strictly between pin and thrum flower, which results in low yield. Improving the yield stability and achene set rate requires a better understanding of the molecular basis of the heteromorphic SI and the development of distylous flowers in common buckwheat. Recent studies suggested that *Primula GLO2*, a *PI*-like MADS-box gene, is identified as an S-linked gene with its expression specific to S-morph flowers and is a strong candidate for the gene controlling anther height, but exactly how it regulates anther height is unclear [4,5].

The *GLOBOSA (GLO)-/PISTILLATA (PI)* like genes originated by duplication in the ancestral B gene of all extant angiosperms [6]. In almost all core eudicots, the *PI*-like genes work together with *APETALA3 (AP3)-/DEFICIENS (DEF)*-like genes to specify proper petal and stamen identity during flower development; both genes encode B-class MADS-box transcription factors which are functional as heterodimers with each other [7,8]. However, the flexibility of *DEF/AP3*- and *GLO/PI*-like protein interactions observed in early-diverging angiosperms may be one reason resulting in highly diverse flower morphologies in these species [7]. *F. esculentum* (Polygonaceae) belongs to the order Caryophyllales (an early-diverging core eudicots clade) and produces distylous flowers with single-whorl showy tepals, representing an obvious difference with most core eudicots flowers, which make it an ideal model for studying floral organ development and evolution [3,9].

Here, the genomic DNA of two *PI*-like MADS-box genes, *FaesPI_1* and *FaesPI_2*, and their corresponding promoters were isolated from common buckwheat. Sequences alignment indicated that both genes show high identity in exon, but obvious differences in intron length and sequence. Furthermore, the promoters of *FaesPI_1* and *FaesPI_2* also found remarkable differences in length and distribution of cis-regulatory elements. A previous study indicated that *FaesPI_1* is required only in stamen identity in *F. esculentum* [10]. In this study, the functional divergence of two common buckwheat *PI*-like genes was explored by analyzing their expression pattern, characterizing their promoters functions, and assessing the complementation phenotype of *pFaesPI_1::FaesPI_1* and *pFaesPI_2::FaesPI_2* transgenic *pi-1 Arabidopsis*. In addition, the possible roles of *FaesPI_1* and *FaesPI_2* genes regulating stamen development of distylous flowers were proposed in common buckwheat. The findings provide clues for understanding the structure and function evolution of *GLO-/PI*-like genes during early-diverging core eudicots.

## 2. Results

### 2.1. Isolation and Characterization of FaesPI_1 and FaesPI_2 from F. esculentum

**Figure 1:**
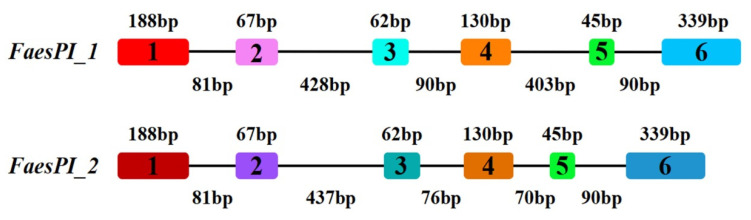
Exon-intron structures of *FaesPI_1* and *FaesPI_2* gene*s*. Color boxes present exons while black lines present introns.

**Figure 2:**
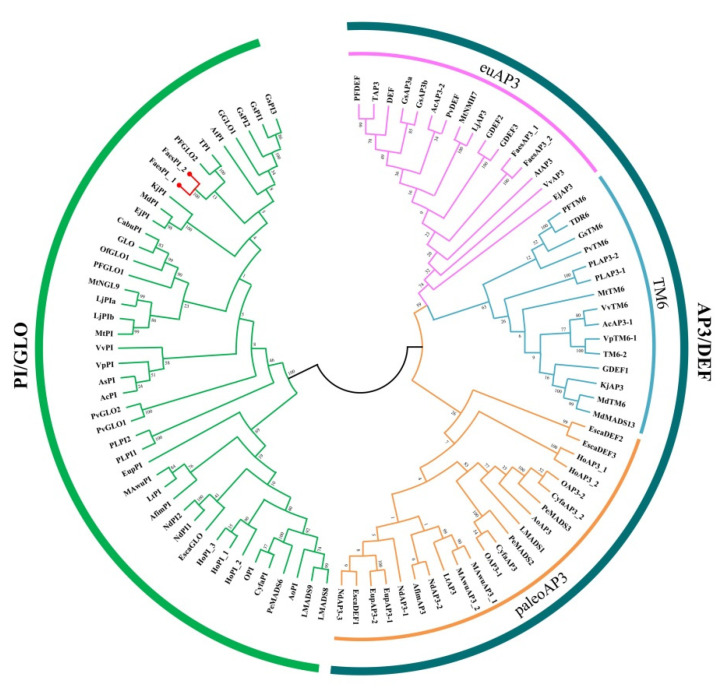
Phylogenetic tree of *FaesPI_1*, *FaesPI_2*, and other B-class MADS-box proteins from different species.

**Figure 3:**
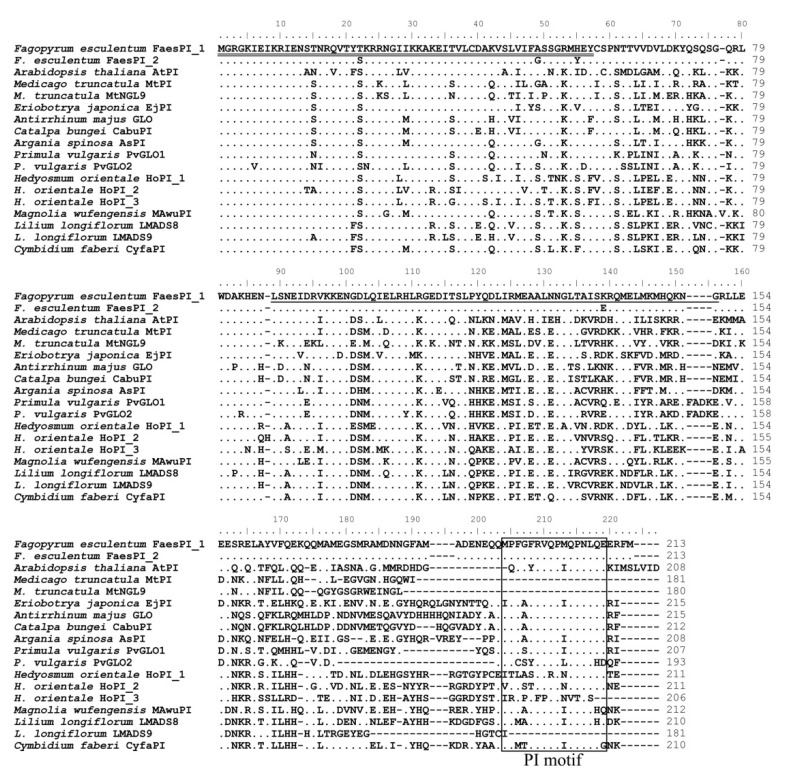
Sequence alignments of *FaesPI_1* and *FaesPI_2* with other PI-like transcription factors (TF) of different species. The double underline refers to the MADS domain and the single underline the refers to K domain. The PI-derived motif lying at the variable C-terminal region is boxed. The dots refer to identical aa with *FaesPI_1*. Moreover, the dashes are introduced into sequences to improve the alignment.

The genomic DNA sequence of *FaesPI_1* (Genbank accession number: OM032616.1) is 1923 bp long and consists of six exons and five introns, while the genomic DNA sequence of *FaesPI_2* (Genbank accession number: OM032617.1) is 1585 bp long and consists of six exons and five introns. Sequences alignment indicated that three introns (the second, the third, and the fourth) of *FaesPI_1* and *FaesPI_2* showed a remarkable difference in sequence and length. However, the sequence and length of the corresponding exon between *FaesPI_1* and *FaesPI_2* showed high conservation (Figure 1). For example, the CDS of *FaesPI_1* and *FaesPI_2* showed 98.18% identity and encoded 213 aa with 98.12% identity. Phylogenetic tree analysis grouped *FaesPI_1* and *FaesPI_2* into *PI/GLO* lineage of B-class MADS-box transcription factor (Figure 2), and both genes were separately designated as *FaesPI_1* (*Fagopyrum esculentum PISTILLATA_1*) and *FaesPI_2*. In addition, proteins alignment shows that each buckwheat *PI*-like transcription factor comprises a 57 aa highly conserved MADS-box domain (1-57), a 64 aa weakly conserved K domain (87-50), and a highly conserved PI motif (194-209) lying at a variable C-terminal region (151-213) (Figure 3) [11].

### 2.2. Expression Analysis of FaesPI_1 and FaesPI_2

**Figure 4:**
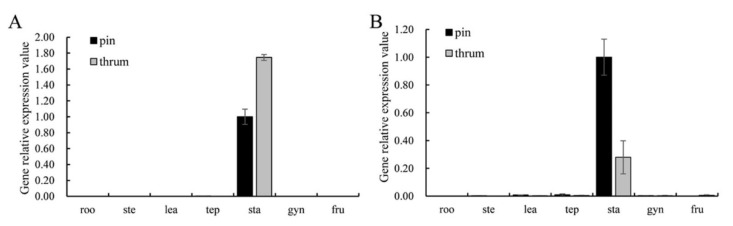
Expression of *FaesPI_1* and *FaesPI_2* in the root (roo), stem (ste), juvenile leaf (lea), tepal (tep), stamen (sta), gynoecium (gyn), and 6-day-old fruits (fru) were detected by qRT-PCR by using *Faesactin* as the internal control. (**A**) *FaesPI_1* expression in seven organs of *F. esculentum*; (**B**) *FaesPI_2* expression in seven organs of *F. esculentum*.

**Figure 5:**
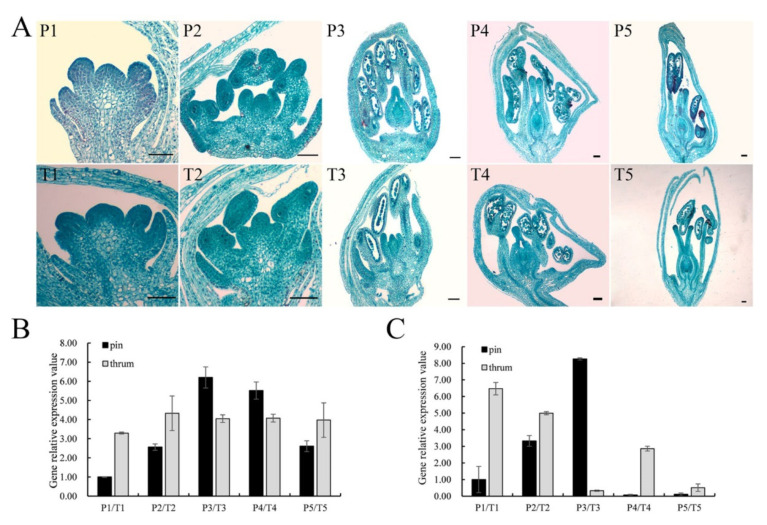
Morphology, *FaesPI_1,* and *FaesPI_2* expression in different development stage distylous flowers of *F. esculentum*. (**A**) Cytomorphological section of thrum and pin floral buds at different development stages; P1–P5: pin floral bud differentiation; P1: stamen primodium appearance; P2: filament rapid elongating; P3: microspore tetrad formation; P4: mononuclear microspore and tepal enclosing; P5: maturity floral bud with mature pollen and embryo sac before bloom; T1–T5: Cytomorphological section of the thrum floral buds; T1: stamen primodium appearance; T2: filament elongation; T3: meiosis of microspore mother cells; T4: periphery of mononuclear microspore, tepal enclosing; T5: maturity floral buds with mature pollen and embryo sac before bloom; (**B**) *FaesPI_1* expression at pin and thrum floral buds were separately detected by qRT-PCR during floral bud differentiation; (**C**) *FaesPI_2* expression at pin and thrum floral buds were separately detected by qRT-PCR during floral bud differentiation. Scale bar: 100 μm.

*FaesPI_1* and *FaesPI_2* were expressed only in the stamen of pin and thrum flower. However, the expression level of *FaesPI_1* in thrum stamen was significantly higher than that of the pin stamen (*p* < 0.01). However, the expression level of *FaesPI_2* in pin stamen was significantly higher than that of the thrum stamen (*p* < 0.05) (Figure 4A,B). *FaesPI_1* and *FaesPI_2* transcripts were detected after stamen primodium emergence in pin and thrum floral buds (Figure 5A–C). Moreover, *FaesPI_1* expression increased constantly and achieved the peak until the microspore tetrad formation (Figure 5A,B(P3)) occurs in the pin flower, and then began to slowly decline until flower maturity. In addition, the *FaesPI_1* expression level when the microspore tetrad formed (Figure 5A,B(P3)) or mononuclear microspore and tepal enclosed (Figure 5A,B(P4)) was significantly higher than that of its expression in other stage floral buds of pin flower (*LSD*, *p* < 0.05). However, *FaesPI_1* expression reached a high level at the filament rapid elongation stage (Figure 5A,B(T2)) in the thrum flower and maintained at a high level until flower maturity. However, *FaesPI_2* expression increased constantly and reached the peak until the microspore tetrad formation (*LSD*, *p* < 0.05) (Figure 5A,C(P3)) in the pin flower, and then there was a sharp drop, which was maintained at a very weak level until flower maturity (Figure 5A,C(P3)). Moreover, *FaesPI_2* expression decreased constantly and reached the bottom until microspore mother cells begin to meiosis (Figure 5A,C(T3)) in the thrum flower, and then maintained at a very low level until flower maturity (Figure 5A,C(T3)).

### 2.3. Characterization of FaesPI_1 and FaesPI_2 Promoters from F. esculentum

A 2.2 kb *FaesPI_1* promoter *(pFaesPI_1)* (-2186/+68) (Genbank accession number: OM032614.1) and a 2.1kb *FaesPI_2* promoter *(pFaesPI_2)* (-2057/+68) (Genbank accession number: OM032615.1) were separately cloned from common buckwheat. The putative cis-acting elements and transcription start site (TSS) of *pFaesPI_1* and *pFaesPI_2* were separately displayed in Figures S1 and S2. The *pFaesPI_1* has a key CArG-box motif (-1231/-1222) for MADS-box TF recognizing and binding [12], and a CArG-box motif (-154/-145) is also found in the *pFaesPI_2*. Moreover, *pFaesPI_1* has eight POLLEN1LELAT52-boxes and three GTGANTG10-boxes, which are cis-regulatory elements usually found in the promoter region of stamen-development regulated genes [13,14], while *pFaesPI_2* contains eleven POLLEN1LELAT52-boxes and nine GTGANTG10-boxes. Moreover, *pFaesPI_1* and *pFaesPI_2* separately contains several AACAAA-/TTTGTT- motifs for floral homeotic protein APETALA2 recognizing and acting [15]. Furthermore, several MYCCONSENSUSAT-boxes are lying at *pFaesPI_1* and *pFaesPI_2*, which indicates that the expression of both genes could be induced by freezing [16]. Some gibberellin-responsive elements are also lying at *pFaesPI_1* (WRKY71OS-box, MYBGAHV-box, and PYRIMIDINEBOXOSRAMY1A-box) and *pFaesPI_2* (WRKY71OS-box) [17,18,19]. Several esophyll-specific elements CACTFTPPCA1-boxes and a UP2ATMSD cis-element associated with gene expression during initiation of axillary bud outgrowth are separately found in *pFaesPI_1* and *pFaesPI_2*, which indicated that the expression of both genes may extend to bud, leaf, and rachis [20,21]. However, two CONSTANS protein binding sites (CCAATBOX1) associated with flowering are only found in *pFaesPI_1* [22], while a target binding site (LEAFYATAG-box) for regulators of floral identity LEAFY (LFY) associated with floral organ development is only lying at *pFaesPI_2* [23]. In addition, four TCP-domain protein-binding elements (SITEIIATCYTC-box) are only found in *pFaesPI_2* [24]. All of these suggested that *pFaesPI_1* and *pFaesPI_2* may drive the corresponding gene to regulate flowering and floral organ development in a different way.

**Figure 6:**
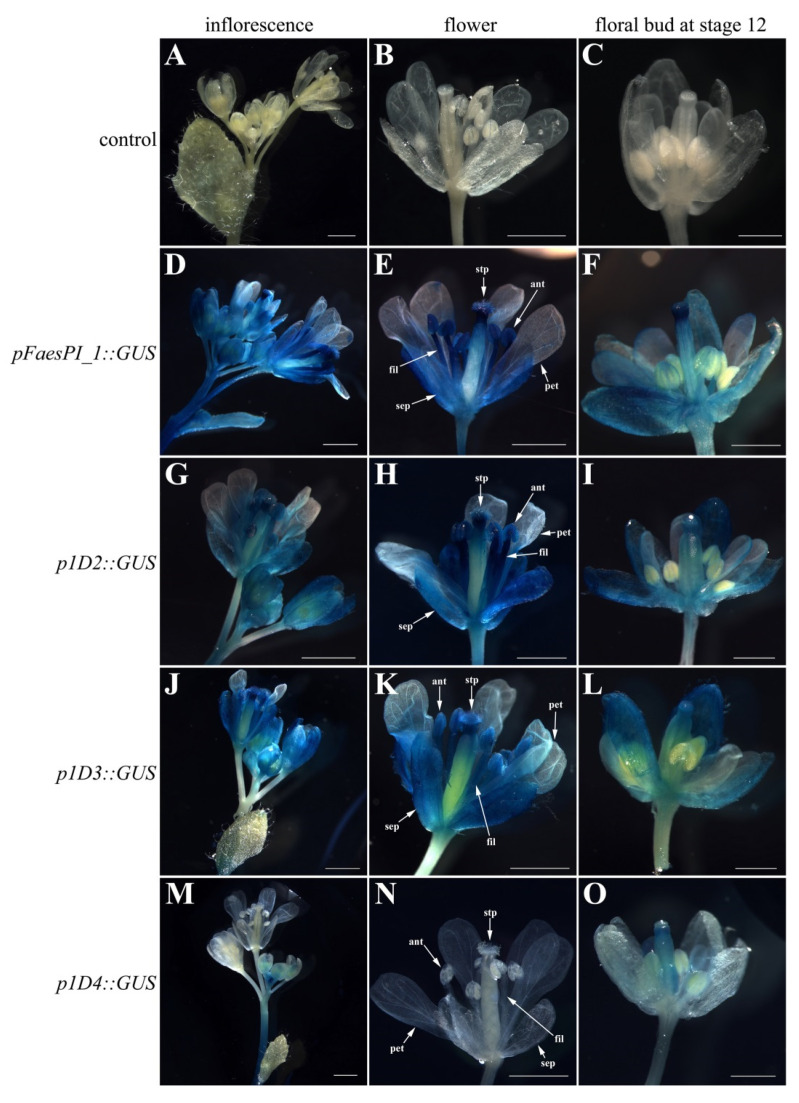
Histochemical GUS staining in the T1 generation of *pFaesPI_1::GUS* transgenic *Arabidopsis* and deletion analysis of the *pFaesPI_1* promoter. (**A**) wild-type *Arabidopsis* Inflorescence; (**B**) wild-type *Arabidopsis* flower; (**C**) stage 12 floral bud of wild-type *Arabidopsis* [25]; (**D**) inflorescence of *pFaesPI_1::GUS* transgenic *Arabidopsis*; (**E**) mature flower of *pFaesPI_1::GUS* transgenic *Arabidopsis*; (**F**) stage 12 floral bud of *pFaesPI_1::GUS* transgenic *Arabidopsis*; (**G**) inflorescence of *p1D2::GUS* transgenic *Arabidopsis*; (**H**) mature flower of *p1D2::GUS* transgenic *Arabidopsis*; (**I**) stage 12 floral bud of *p1D2::GUS* transgenic *Arabidopsis*; (**J**) inflorescence of *p1D3::GUS* transgenic *Arabidopsis*; (**K**) mature flower of *p1D3::GUS* transgenic *Arabidopsis*; (**L**) stage 12 floral bud of *p1D3::GUS* transgenic *Arabidopsis*; (**M**) inflorescence of *p1D4::GUS* transgenic *Arabidopsis*; (**N**) mature flower of *p1D4::GUS* transgenic *Arabidopsis*; (**O**) stage 12 floral bud of *p1D4::GUS* transgenic *Arabidopsis*. sepal (sep), petal (pet), anther (ant), filament (fil), stigmatic papillae (stp); Scale Bars: (**A**,**B**,**D**,**E**,**G**,**H**,**J**,**K**,**M**,**N**) 1 mm; (**C**,**F**,**I**,**L**,**O**) 500 μm.

**Figure 7:**
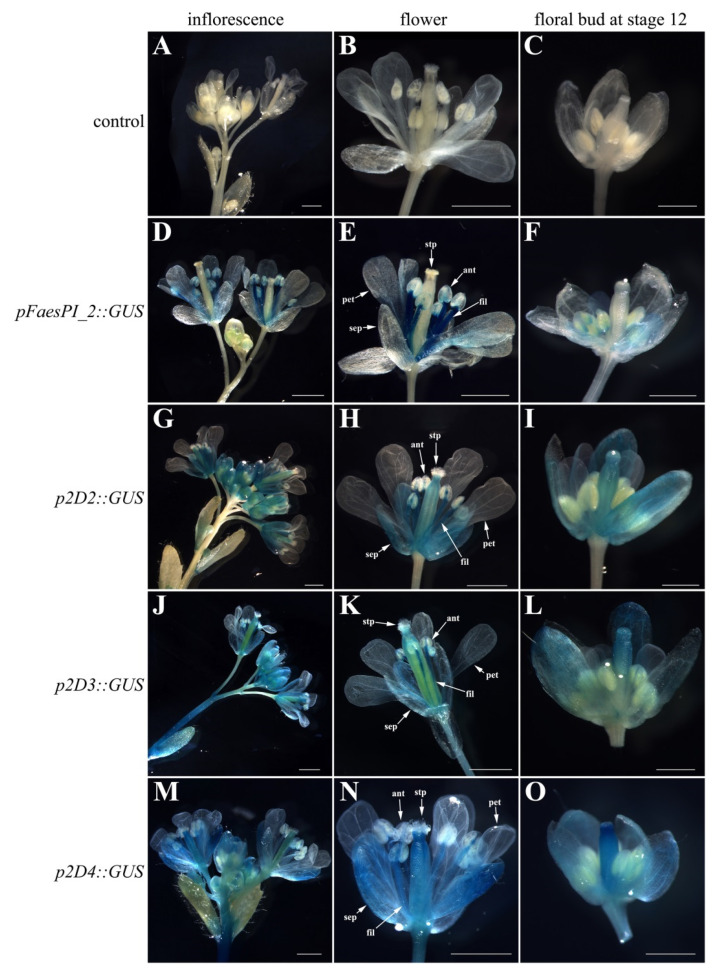
Histochemical GUS staining in the T1 generation of *pFaesPI_2::GUS* transgenic *Arabidopsis* and deletion analysis of the *pFaesPI_2* promoter. (**A**) wild-type *Arabidopsis* Inflorescence; (**B**) wild-type *Arabidopsis* flower; (**C**) stage 12 floral bud of Wild-type *Arabidopsis*; (**D**) inflorescence of *pFaesPI_2::GUS* transgenic *Arabidopsis;* (**E**) mature flower of *pFaesPI_2::GUS* transgenic *Arabidopsis;* (**F**) stage 12 floral bud of *pFaesPI_2::GUS* transgenic *Arabidopsis;* (**G**) inflorescence of *p2D2::GUS* transgenic *Arabidopsis;* (**H**) mature flower of *p2D2::GUS* transgenic *Arabidopsis;* (**I**) stage 12 floral bud of *p2D2::GUS* transgenic *Arabidopsis;* (**J**) inflorescence of *p2D3::GUS* transgenic *Arabidopsis;* (**K**) mature flower of *p2D3::GUS* transgenic *Arabidopsis;* (**L**) stage 12 floral bud of *p2D3::GUS* transgenic *Arabidopsis;* (**M**) inflorescence of *p2D4::GUS* transgenic *Arabidopsis;* (**N**) mature flower of *p2D4::GUS* transgenic *Arabidopsis;* (**O**) stage 12 floral bud of *p2D4::GUS* transgenic *Arabidopsis*. sepal (sep), petal (pet), anther (ant), filament (fil), stigmatic papillae (stp); Scale Bars: (**A**,**B**,**D**,**E**,**G**,**H**,**J**,**K**,**M**,**N**) 1 mm; (**C**,**F**,**I**,**L**,**O**) 500 μm.

A *beta-glucuronidase* (*GUS*) reporter gene separately driven by *pFaesPI_1* and *pFaesPI_2* was examined in transgenic *Arabidopsis* (Figure 6 and Figure 7). GUS staining was separately examined in the T1 generation of *pFaesPI_1::GUS* and *pFaesPI_2::GUS* independent transgenic lines. *GUS* expression was found in the inflorescence and flower where sepal, filament, anther, stigma, and stigmatic papillae were high, but was absent in a petal of *pFaesPI_1::GUS* transgenic *Arabidopsis* (Figure 6D,E). Moreover, *GUS* expression was observed in the stage 12 floral bud where sepal, filament, stigma, and stigmatic papillae were intensive, but was absent in petal and anther of *pFaesPI_1::GUS* transgenic *Arabidopsis* (Figure 6F) [25]. However, *GUS* expression was obviously observed in the filament of a mature flower but was almost absent in the sepal, petal, anther, and gynoecium of *pFaesPI_2::GUS* transgenic *Arabidopsis* (Figure 7D,E). Moreover, weak *GUS* expression was observed only at the anther-filament junction in the stage 12 floral bud of *pFaesPI_2::GUS* transgenic *Arabidopsis* (Figure 7F).

### 2.4. Deletion Analysis of the pFaesPI_1 and pFaesPI_2 in Transgenic Arabidopsis

A series of *5′* deletions fragments of the *pFaesPI_1* and *pFaesPI_2* were separately fused to the *GUS* gene and transformed into *Arabidopsis* to analyze the regulatory effect of different regions of the corresponding promoter. GUS staining suggested that p1D2 (-1402/+68) and p1D3 (-817/+68) constructs presented similar expression patterns with the *pFaesPI_1::GUS* transgenic *Arabidopsis,* which was high in the inflorescence, sepal, stamen, stigma, and stigmatic papillae of a mature flower (Figure 6). In addition, further deletion of the -817/-366 fragment from p1D3 to produce p1D4 (-365/+68) caused obviously decreased GUS activity in transgenic *Arabidopsis*, and weak GUS staining was only observed in the gynoecium of early development floral buds from initiation until stage 12 (Figure 6). These results suggested that the -1402/-366 regions are capable of inducing *pFaesPI_1* promoter activity in stamen, stigma, and stigmatic papillae, and an 885 bp region (-817/+68) of *pFaesPI_1* was sufficient for driving *FaesPI_1* gene to regulate stamen development. However, GUS staining suggested that p2D2 (-1532/+68), p2D3 (-1032/+68), and p2D4 (-250/+68) constructs presented similar expression zones, which were only in sepal, filament, and the gynoecium of transgenic *Arabidopsis*, and showed different expression zones with the *pFaesPI_2::GUS* transgenic *Arabidopsis* (Figure 7). Moreover, intensive GUS staining was found only in the filament of *pFaesPI_2::GUS* transgenic *Arabidopsis*. The results also indicated that the -2057/-1532 regions contained regulatory elements critical for restricting *FaesPI_2* expression to the filament (Figure 7E).

### 2.5. Phenotypic Analyses of pFaesPI_1::FaesPI_1 and pFaesPI_2::FaesPI_2 Transgenic pi-1 Arabidopsis

To uncover the roles of FaesPI_1 and FaesPI_2 involved in floral development, pFaesPI_1::FaesPI_1, and pFaesPI_2::FaesPI_2 constructs have been separately transformed into PI/pi-1 heterozygote Arabidopsis to create phenotype complementation lines. All transgenic plants were verified by qRT-PCR. In addition, the independent transgenic lines of pFaesPI_1::FaesPI_1 or pFaesPI_2::FaesPI_2 Arabidopsis under wild-type, heterozygote and homozygous background were verified by using dCAPS method with BspHI (TaKaRa Bio, Otsu, Japan) restriction enzymes, respectively (Supplementary Figure S3). Moreover, FaesPI_1 and FaesPI_2 expression in transgenic lines under homozygous backgrounds were separately detected. In addition, 11 independent pFaesPI_1::FaesPI_1 lines under homozygous pi-1 mutant background and 21 independent pFaesPI_2::FaesPI_2 lines under homozygous pi-1 mutant background were obtained, respectively. Flower phenotypes of each transgenic line after flowering were assessed to evaluate whether FaesPI_1 or FaesPI_2 could replace the endogenous PI gene to control petal and stamen development in Arabidopsis pi-1 mutant.

**Figure 8:**
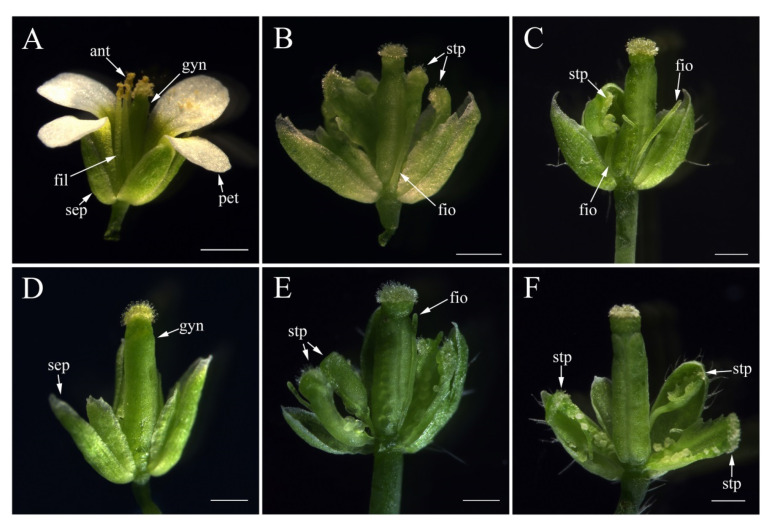
Flower phenotypes of wild-type, *Arabidopsis pi-1* mutant, *pFaesPI_1::FaesPI_1* transgenic *pi-1 Arabidopsis,* and *pFaesPI_2::FaesPI_2* transgenic *pi-1 Arabidopsis*. (**A**) wild-type *Arabidopsis* flower with perfect flower (sepal in the first floral whorl, petal in the second floral whorl, stamen in the third floral whorl and fused carpel in the fourth floral whorl); (**B**) flower of *pFaesPI_1::FaesPI_1* transgenic homozygous *pi-1 Arabidopsis* with carpelloid stamen or filament with stigmatic papillae at the top in the third floral whorl; (**C**) flower of *pFaesPI_1::FaesPI_1* transgenic homozygous *pi-1 Arabidopsis* with filament and filament with carpelloid anther at the top in the third floral whorl; (**D**) flower of *Arabidopsis pi-1* mutant exhibits homeotic transformations of the second floral whorl petal to sepal, and stamen deficiency in the third floral whorl; (**E**) flower of *pFaesPI_2::FaesPI_2* transgenic homozygous *pi-1 Arabidopsis* with carpelloid stamen and filament-like organ in the third floral whorl; (**F**) flower of *pFaesPI_2::FaesPI_2* transgenic homozygous *pi-1 Arabidopsis* with carpelloid stamen and filament with carpelloid anther at the top in the third floral whorl. sepal (sep), petal (pet), anther (ant), filament (fil), filament-like organ (fio), stamen-like organ (sto), gynoecia (gyn), stigmatic papillae (stp). Scale Bars: 500 μm.

Among eleven *pFaesPI_1::FaesPI_1* transgenic *pi-1 Arabidopsis,* eight (72.73%) showed stamen complementation phenotypes with carpelloid stamen, filament-like organs, or filament with carpelloid anther or stigmatic papillae at the top in the third whorl of the flower (Figure 8B,C), the remaining three (27.27%) lines showed similar flower phenotypes with homozygous *pi-1 Arabidopsis*. Among twenty-one *pFaesPI_2::FaesPI_2* transgenic *pi-1 Arabidopsis,* nineteen (90.48%) showed a graded complementation phenotype with carpelloid stamen, filament-like organs, or filament with carpelloid anther at the top in the third whorl of the flower (Figure 8E,F), other three (9.52%) lines showed similar flower phenotype with homozygous *pi-1 Arabidopsis*.

## 3. Discussion

In core eudicots, most *GLO*-/*PI*-like genes, such as *EjPI* from *Eriobotrya japonica* [26], *GGLO1* from *Gerbera hybrida* [27], and *AsPI* from *Argania spinosa* [28], were expressed in the petal and stamen and were mainly required in controlling perfect petal and stamen identities during flower development. The data indicate that the functions of *GLO*-/*PI*-like genes are highly correlaTaKaRa, Shigated with their expression zones in core eudicots. However, many *GLO*-/*PI*-like genes usually displayed broader expression zones and versatile functions in basal angiosperms, basal eudicots, and monocots. For example, *Hedyosmum orientale PI*-like genes *HoPI* were broadly expressed in all floral organs but were involved only in specifying petal and stamen identities [29]. *Magnolia wufengensis PI* orthologous gene, *MAwuPI,* was expressed in petaloid tepal and stamen but was required only for stamen identity [30]. Lily *PI* orthologous genes, *LMADS8/9,* were expressed in tepal and stamen and were required for tepal and stamen formation [31,32]. Some orchid *PI-like* genes, such as *OMADS8* from *Oncidium* and *OPI* from *Phalaenopsis*, were found expressed in all floral organs but were required only for specifying perianths (sepal/petal and lip) and androecium formation [33,34,35]. The data indicate that stamen-specific function obtained from *PI* orthologs antedate their petal-specific function during angiosperm evolution. *F. esculentum* is a member of the family Polygonaceae in the order Caryophyllales, a member of an early-diverging clade of higher eudicots, and has distylous flowers without the petal whorl [3,9]. In addition, *F. esculentum PI*-like genes, *FaesPI_1* and *FaesPI_2*, were expressed only in the stamen. The expression absence of *FaesPI_1* and *FaesPI_2* in showy tepals suggested that the *PI-like* gene-dependent petal identity program was not observed in *F. esculentum*. The findings provided new clues for understanding flower variation and interpretation of petal evolution across core eudicots. Moreover, intensive GUS staining was observed in the whole stamen (filament and anther) of *pFaesPI_1::GUS* transgenic *Arabidopsis,* while intensive GUS staining was found only in the filament of *pFaesPI_2::GUS* transgenic *Arabidopsis*. Phenotype complementation analysis suggested that *pFaesPI_1::FaesPI_1*/*pFaesPI_2::FaesPI_2* transgenic *pi-1 Arabidopsis* showed similar flower structure with stamen-like organs or filament-like organs in the third whorl. The data suggest that *FaesPI_2* may be involved only in filament development, and *FaesPI_1* may specify stamen development in common buckwheat.

Small-scale gene duplication events of *GLO-/PI-*like genes happened throughout angiosperms. In addition, most *PI*-like paralogs have undergone functional overlap or subfunctionalization after gene duplication. For example, *NdPI1* *and NdPI2* were two *PI*-like genes from basal eudicots *Nigella damascene* (Ranunculaceae)*. NdPI1* *and NdPI2* were mainly expressed in sepal, petal, and stamen, and have functioned redundantly in specifying petal and stamen identity [36]. Two *GLO/PI* orthologous genes, *PLPI1* and *PLPI2* from basal eudicots *Paeonia lactiflora*, were strongly expressed in petal, stamen, and carpel; *PLPI1* was required for petal and stamen identity, while *PLPI2* was sufficient to guarantee stamen identity [37]. However, few paralogs acquire new functions after duplication. For example, *Medicago truncatula PI-*like gene *MtPI* maintained the overall ancestral function for specifying petal and stamen identity, while another *PI-*like gene *MtNGL9*, may be required to maintain the critical dosage for the B-function in *M. truncatula* [38]. In distylous *Primula*, *GLO/PI* orthologous gene *GLO1* maintained the ancestral B-class function in specifying petal and stamen identity after duplication, while *GLO2* underwent neofunctionalization for promoting the cell expansion in the fused tube of petals and stamen filaments and determining anther position. Moreover, the GLO2 gene at the S locus worked together with the style-length-determining gene *CYP734A50* for the development of heterostyly [4,39]. In our study, *F. esculentum pFaesPI_2* could drive the *GUS* gene to be expressed only in the filament of transgenic *Arabidopsis*, while *pFaesPI_1* could drive the *GUS* gene to be expressed in the whole stamen (filament and anther) of transgenic *Arabidopsis*. A previous study also proved that the *GLO/PI-*like orthologs and *AP3/DEF* usually work together to specify perfect petal and stamen identity during flower development in almost all core eudicots [7,8]. Our previous study also suggested that two *AP3-like* paralogs, *FaesAP3_1* and *FaesAP3_2,* had functioned redundantly in controlling filament identity, and *FaesAP3_2* had a key role in regulating anther development [40]. The data suggest that *FaesPI_2* may interact with *FaesAP3_1* to filament development during common buckwheat flower development. In addition, a future challenge was to explore whether the *FaesPI_2* works together with the candidate S locus gene to regulate heterostyly in *F. esculentum*. Our data suggest the functional divergences of *PI*-like paralogs after duplication in an early-diverging clade of core eudicots, and also provide an idea candidate gene for the potential application in bioengineering to develop a common buckwheat male sterile line.

## 4. Materials and Methods

### 4.1. Plant Material

Floral buds at various developmental stages were sampled from thrum and pin plants of buckwheat ‘Beizaosheng’ planted under natural conditions in Jingzhou, Hubei Provence, China, respectively. Moreover, each sample was divided in half; one was immediately frozen in liquid nitrogen and then stored at −80 °C, and another was incubated in FAA [38% formaldehyde: acetic acid: 70% ethanol = 1:1:18 (V/V)]. The roots, stems, juvenile leaves, tepals, stamens, gynoecia and 6-day-old fruits (achenes) of thrum and pin plants were dissected, respectively, and were immediately frozen and stored in liquid nitrogen. The *Arabidopsis pi-1* mutant (CS77) seeds were obtained from the *Arabidopsis* Biological Resource Center (ABRC) at Ohio State University, USA.

### 4.2. Characterization of Genomic DNA FaesPI_1 and FaesPI_2 from F. esculentum

Buckwheat genomic DNA was extracted from juvenile leaves of thrum and pin plants using the CTAB Plant Genomic DNA Rapid Extraction Kit (Aidlab, Beijing, China) referring to the manufacturer’s protocol. The full-length genomic DNA sequences of *FaesPI_1* and *FaesPI_2* were separately isolated from *F. esculentum* genomic DNA with a forward primer DFaesPIF and reverse primer DFaesPIR (Supplementary Table S1), and then were cloned into the pTOPO-TA vector (Aidlab, Beijing, China) for sequencing, respectively. The PCR primers were designed based on the buckwheat *PI*-like gene (Genbank accession numbers: JN605356.1) identified before. The PCR amplification of *FaesPI_1* or *FaesPI_2* genomic DNA was carried out in a 25 µL reaction volumes containing 4 µL dNTP Mixture (2.5 mM each) (TaKaRa Bio, Otsu, Japan), 2.5 µL 10 × LA PCR Buffer II (Mg^2+^ plus), and 0.3 µL LA Taq DNA Polymerase (5 U/µL) (TaKaRa Bio, Otsu, Japan). PCR was carried out with denaturation at 94 °C (3 min), followed by 30 cycles of 30 s at 94 °C, annealing at 58 °C (30 s), extension at 72 °C (90 s), with a final extension period (10 min). Phylogenetic tree construction was performed by using the neighbor-joining (NJ) method in MEGA version 5.05. The NJ tree was built with the Poisson model and 1000 bootstrap replications. All the B-class MADS-box transcription factors (TF) sequences containing whole M, I, K, and C domains were obtained from NCBI Genbank (Supplementary Table S2). In order to characterize FaesPI_1 and FaesPI_2 in detail, the TF sequence alignment was aligned by the ClustalW algorithm with 1000 bootstrap replications in BioEdit version 7.0.9. Pairwise alignment was carried out with a gap opening penalty of 10 and a gap extension penalty of 0.1, and multiple alignments were performed with a gap opening penalty of 10 and a gap extension penalty of 0.2.

### 4.3. Isolation and Sequence Analysis of FaesPI_1 and FaesPI_2 Promoters from F. esculentum

The *FaesPI_1* 5′ flanking regions were cloned using the method suggested by Liu et al. [41], but with three reverse primers D1pPISP1, D1pPISP2, and D1pPISP3 for the walking sequencing. The *FaesPI_2* 5′ flanking regions were coloned following the above method, but with three reverse primers D1pPISP1, D1pPISP2, and D1pPISP3 for the first walking sequencing, and with three reverse primers D2pPI_2SP1, D2pPI_2SP2, and D2pPI_2SP3 for the second walking sequencing. Moreover, the full-length *FaesPI_1* promoter (*pFaesPI_1*) was amplified via PCR and cloned into the pTOPO-TA vector (Aidlab, Beijing, China) with the forward primer TpFaesPI_1F and the reverse primer TpFaesPI_1R for sequencing. The full-length *FaesPI_2* promoter (*pFaesPI_2*) was amplified via PCR and cloned into the pTOPO-TA vector (Aidlab, Beijing, China) with the forward primer TpFaesPI_2F and the reverse primer TpFaesPI_2R for sequencing. In addition, the putative transcription start site of *FaesPI_1* or *FaesPI_2* was searched by using the 5′RACE method with the 5′RACE System for Rapid Amplification of cDNA Ends (Invitrogen, Carlsbad, CA, USA) referring to the manufacturer’s protocol, and three gene-specific reverse primers 5RPI_1GSP1, 5 RPI_1GSP2 and 5 RPI_1GSP3 for *FaesPI_1*, but three gene-specific reverse primers 5RPI_2GSP1, 5 RPI_2GSP2 and 5 RPI_2GSP3 for *FaesPI_2*. The cis-acting regulatory elements of the *pFaesPI_1* and *pFaesPI_2* promoters were found in the PLACE database [42].

### 4.4. Characterization of pFaesPI_1 and pFaesPI_2 Activity from the 5′ Deleted Promoter Fragments in Transgenic Arabidopsis

Four forward primers (TpFaesPI_1F, TpFaesPI_1F1, TpFaesPI_1F2, and TpFaesPI_1F3) and a reverse primer TpFaesPI_1/2R were designed to obtain 5′-deletion fragments of *pFaesPI_1*. Moreover, four forward primers (TpFaesPI_2F, TpFaesPI_2F1, TpFaesPI_2F2, and TpFaesPI_2F3) and reverse primer TpFaesPI_1/2R were designed to obtain 5′-deletion fragments of *pFaesPI_2*. Four 5′-deletion fragments of *pFaesPI_1* were designated as p1D1 (-2186/+68),p1D2 (-1402/+68),p1D3 (-817/+68), and p1D4 (-365/+68), and were separately cloned into the pCAMBIA1300 with *Xba* I (TaKaRa Bio, Otsu, Japan) and *Sac* I (TaKaRa Bio, Otsu, Japan) restriction enzymes using the ClonExpress^®^ Ultra One Step Cloning Kit (Vazyme, Nanjing, China) following the manufacturer’s protocol. Moreover, four 5′-deletion fragments of *pFaesPI_2* were designated as p2D1 (-2057/+68), p2D2 (-1532/+68), p2D3 (-1032/+68), and p2D4 (-250/+68), and were separately cloned into the pCAMBIA1300 vector using the above method. All the constructs were separately transformed into *A. thaliana Col-0* plants using the floral-dip method according to Clough and Bent [43]. Transgenic *Arabidopsis* seedlings were selected, cultivated, and prepared for histochemical GUS staining using the method suggested by Liu et al. [41].

For GUS staining, the inflorescences of transgenic *Arabidopsis* were incubated in 90% acetone (4 °C for 20 min) and then rinsed with GUS assay buffer [50 mM sodium phosphate (pH 7.0), 1 mM K_3_Fe(CN)_6_, 1 mM K_4_Fe(CN)_6_·3H_2_O, 10 mM EDTA (pH 8.0), 0.2 % Triton X-100 (V/V)] 2–3 times, followed by vacuum infiltrated in a mixture of GUS assay buffer and 2 mM X-Gluc for 30 min at room temperature, and then incubated for 6 h at 37 °C, discarding the liquids and later cleared in an ethanol series (75, 85, 95 and 100%). The samples were observed with a Leica 165C microscope (Leica Microsystems, Wetzlar, Germany), and the photomicrographs were taken.

### 4.5. Cytomorphological Observation and Expression Analysis of FaesPI_1 and FaesPI_2

The floral bud samples of thrum and pin plants fixed in FAA above were separately dehydrated using an ethanol series, cleared twice in xylene, infiltrated three times in molten paraffin, embedded into paraffin block, serially sectioned, and then sections were separately stained according to the method described by Liu et al. [41]. Each section was observed under a CAIKON RCK-40C microscope (CAIKON, Shanghai, China) and the Photomicrographs were taken.

Total RNA and the first-strand cDNA of each sample were prepared for quantitative real-time PCR (qRT-PCR) according to Zeng et al. [40]. The expressions of *FaesPI_1* and *FaesPI_2* were separately detected in seven organs (root, stem, juvenile leaf, tepal, stamen, gynoecium, and 6-day-old fruit) of different flower phenotype plants according to Zeng et al. [40], but with the primers qFaesPI_1F and qFaesPI_1R for *FaesPI_1,* and the primers qFaesPI_2F and qFaesPI_2R for *FaesPI_2*. In addition, *FaesPI_1* and *FaesPI_2* expressions were separately detected in thrum and pin floral buds at sequential developmental stages using qRT-PCR suggested above. The amplicons of *F. esculentum actin* gene (Genbank accession number: HQ398855.1) were selected as the internal control with the forward primer qFaesactinF and the reversed primer qFaesactinR. qRT-PCR was performed with three biological replicates and the relative expression levels were measured according to Liu et al. [41], but with annealing at 58 °C.

### 4.6. Phenotypic Analyses of pFaesPI_1::FaesPI_1 and pFaesPI_2::FaesPI_2 Transgenic pi-1 Arabidopsis

Full-length *pFaesPI_1::FaesPI_1* genomic DNA was cloned into pCAMBIA1300 with *Xba* I (TaKaRa Bio, Otsu, Japan) and *Sac* I (TaKaRa Bio, Otsu, Japan) restriction enzymes, and the primer pairs Tp1DFaesPI_1F and TpDFaesPIR using the ClonExpress^®^ Ultra One Step Cloning Kit (Vazyme, Nanjing, China) following the manufacturer’s protocol. Meanwhile, full-length *pFaesPI_2::FaesPI_2* genomic DNAs were cloned into the pCAMBIA1300 vector with the above method, but the forward primer Tp2DFaesPI_2F and the reverse primer TpDFaesPIR. The *pFaesPI_1::FaesPI_1* and *pFaesPI_2::FaesPI_2* constructs were separately transformed into *PI*/*pi-1* heterozygote *Arabidopsis* through the floral-dip suggested by Clough and Bent [43]. Transgenic *Arabidopsis* seedlings were screened, cultivated, and identified referring to Fang et al. [10]. Homozygous *pi-1* transgenic *Arabidopsis* lines were obtained using the dCAPS genotyping method described by Lamb and Irish [44]. Phenotypes of all transgenic *Arabidopsis* lines were separately assessed after flowering.

Moreover, the phenotype complementation degrees of independent transgenic lines of *pFaesPI_1::FaesPI_1* or *pFaesPI_2::FaesPI_2* homozygous *pi-1 Arabidopsis* were classified as ‘no complementation’, ‘medium complementation’, and ‘strong complementation’, respectively. In addition, independent transgenic lines of each complementation degree were verified by qRT-PCR according to the above method, but with the primers qTFaesPI_1F and qTFaesPI_1R for *FaesPI_1,* and with the primers qTFaesPI_2F and qTFaesPI_2F for *FaesPI_2*. Amplification fragment of *A. thaliana Actin* (Genbank accession numbers: AY114679.1) with the primers qActinF and qActinF was the internal control.

## 5. Conclusions

*F. esculentum* (Polygonaceae) belongs to the order Caryophyllales (an early-diverging core eudicots clade) and produces distylous flowers with single-whorl undifferentiated showy tepals, representing an obvious difference with flowers of most core eudicots, which makes it an ideal model for exploring floral organ development and evolution. The *Arabidopsis* floral homeotic B-class MADS-box gene *PISTILLATA* (*PI*) is expressed in petal and stamen and works together with another B-function gene *APETALA3* (*AP3*) to specify petal and stamen identity. However, a small-scale gene duplication (GD) event was found in the common buckwheat *PI* ortholog and resulted in *FaesPI_1* and *FaesPI_2*. Furthermore, *FaesPI_1*/*2* were expressed only in the stamen of the distylous flower. The expression absence of *FaesPI_1*/2 in showy tepals suggested that *the PI*-like gene-dependent petal identity program was not observed in *F. esculentum*. In addition, *GUS* driven by *pFaesPI_1* promoter was expressed in the whole stamen of *pFaesPI_1::GUS* transgenic *Arabidopsis*, while *GUS* driven by *pFaesPI_2* promoter was expressed only in the filament of stamen in *pFaesPI_2::GUS* transgenic *Arabidopsis*. Moreover, *pFaesPI_1::FaesPI_1*/*pFaesPI_2::FaesPI_2* transgenic *pi-1 Arabidopsis* produced a similar flower with stamen-/filament-like organs in the third whorl. All these suggested that *FaesPI_2* may only specify filament development, but *FaesPI_1* may specify stamen development. Meanwhile, *FaesPI_1* and *FaesPI_2* had overlapping functions in specifying stamen filament identity and working together to regulate normal stamen development.

## Data Availability

All data generated or analyzed during this study are included in this published article.

## Supplementary Materials

The following are available online at https://www.mdpi.com/article/10.3390/plants11081047/s1, Table S1: Primers used in this study. Table S2: Information on Sequences selected for alignments and phylogenetic analyses from NCBI GenBank. Figure S1: *Faes**PI_1* promoter sequence. Figure S2: *Faes**PI_2* promoter sequence. Figure S3: Genotyping of wildtype, heterozygous and homozygous *pi-1* mutant *A. thaliana* by dCAPS.
