# Supplementary material for: Characterization of PISTILLATA-like Genes and Their Promoters from the Distyly Fagopyrum esculentum
Source: Plants (Basel). 2022 Apr 12;11(8):1047. doi: 10.3390/plants11081047 (PMC9032694; doi:10.3390/plants11081047)
Supplement: Supplementary file 1 [file plants-11-01047-s001.zip › plants-1667841-supplementary.pdf]

Supplementary Materials

Table S1. Primers used in this study.

Primer name	Primers sequences (5'-3')
DFaesPIF	ATGGGGAGAGGGAAGATTGAAATCAAG
DFaesPIR	ATCATAGAGTTCAGTTCACACACCCATAG
D1pPISP1	CGTGGTATTAGGGCTACAATACTCA
D1pPISP2	CCAGAACTCGCAAAGATAACCAGAG
D1pPISP3	TGCATCACACAGAACAGTAATCTCC
D2pPI_2SP1	AAATGGGTCGGGTCAAAGGAAGAA
D2pPI_2SP2	TGCAGTCTCCCACCACATCCACATG
D2pPI_2SP3	GATACAAGGGTAAGCGAAGTGGT
5RPI_1GSP1	TCTTGCTTATGGCGGTG
5RPI_1GSP2	GGAGAGAAGTGATGTCTTCTCCTCTTAGATGT
5RPI_1GSP3	GCATCCCACAATCTCTGACC
5RPI_2GSP1	TCTCGCTTATGGCGGTA
5RPI_2GSP2	AATCTGTAAGTCACCATTTTCTTTCTTCACTC
5RPI_2GSP3	GCATCCCACAATCTCTGACC
TpFaesPI_1F1	ATGATTACGAATTCGAGCTCCAGAAATCCTTAACTCCTTCCTTC
TpFaesPI_1F2	ATGATTACGAATTCGAGCTCGACCCTTGGCTAGTATCAAA
TpFaesPI_1F3	ATGATTACGAATTCGAGCTCCTCGAATAGATGCAGGAACT
TpFaesPI_1F4	ATGATTACGAATTCGAGCTCTTTTTGTAGAGAGAAACATGTACG
TpFaesPI_2F1	ATGATTACGAATTCGAGCTCCTCGAAGGTATGAAGGAATA
TpFaesPI_2F2	ATGATTACGAATTCGAGCTCGACGATGAATAAGCGGTTAC
TpFaesPI_2F3	ATGATTACGAATTCGAGCTCTGACAACCTTACGCTAATCA
TpFaesPI_2F4	ATGATTACGAATTCGAGCTCTTTTTGTAGAGAGAAACATGTACG
TpFaesPI_1/2R	TCTGCAGGTCGACTCTAGACTCCAAAAACAAACCCTAGT
Tp1DFaesPI_1F	ATGATTACGAATTCGAGCTCCAGAAATCCTTAACTCCTTCCTTC
Tp2DFaesPI_2F	ATGATTACGAATTCGAGCTCGAAGGTATGAAGGAATATGAC
TpDFaesPIR	TCTGCAGGTCGACTCTAGAATCATAGAGTTCAGTTCACACACC
qGUSF	GTTCTGCGACGCTCACACCGATA
qGUSR	CAGCCATGCACACTGATACTCTTC
qFaesPI_1F	AACCAACAGACAAGTTACTTATA
qFaesPI_1R	ATGCATCCTGCCAGAACTCG
qFaesPI_2F	AACCAACAGACAAGTTACTTATT
qFaesPI_2R	ACATCCTGCCAGAACTCC
qFaesactinF	ACCTTGCTGGACGTGACCTTAC

qFaesactinR	CCATCAGGAAGCTCATAGTTC
qTFaesPI_1F	GGAGATTACTGTTCTGTGTGATGC
qTFaesPI_1R	TGTCCAGAACATCAACAACCGTG
qTFaesPI_2F	TGGTTATCTTTGGGAGTTCTGGC
qTFaesPI_2R	CTTTGCATCCCACAATCTCTGAC
qActinF	GACCGTATGAGCAAAGAGATC
qActinR	CACATCTGTTGGAAGGTGCT

Table S2. Information on Sequences selected for alignments and phylogenetic analyses from NCBI GenBank.

Taxon and species	Protein name	Accession numbers	Lineage
Aristolochiaceae			
Aristolochia fimbriata	AfimPI	ALV83436.1	PI/GLO
	AfimAP3	ALV83437.1	AP3/DEF
Magnoliaceae			
Liriodendron tulipifera	LtPI	AIE44761.1	PI/GLO
	LtAP3	AIE44760.1	AP3/DEF
Magnolia wufengensis	MAwuPI	AFM75882.1	PI/GLO
	MAwuAP3_1	AFM75880.1	AP3/DEF
	MAwuAP3_2	AFM75881.1	AP3/DEF
Chloranthaceae			
Hedyosmum orientale	HoPI_1	AFP17796.1	PI/GLO
	HoPI_2	AFP17799.1	PI/GLO
	HoPI_3	AFP17800.1	PI/GLO
	HoAP3_1	AFP17801.1	AP3/DEF
	HoAP3_2	AFP17802.1	AP3/DEF
Liliaceae			
Lilium longiflorum	LMADS8	AEI88009.1	PI/GLO
	LMADS9	AEI88010.1	PI/GLO
	LMADS1	AAM27456.1	AP3/DEF
Orchidaceae			
Cymbidium faberi	CyfaPI	QEO60647.1	PI/GLO
	CyfaAP3_1	ADI58467.1	AP3/DEF
	CyfaAP3_2	QLJ83030.1	AP3/DEF
Oncidium gower ramsey	OPI	ADJ67236.1	PI/GLO
	OAP3-1	ADJ67234.1	AP3/DEF
	OAP3-2	ADJ67235.1	AP3/DEF
Phalaenopsis equestris	PeMADS6	AAV83997.1	PI/GLO
	PeMADS2	AAR26628.1	AP3/DEF

	PeMADS3	AAR26629.1	AP3/DEF
Zingiberaceae			
Alpinia oblongifolia	AoPI	ABB92623.1	PI/GLO
	AoAP3	ABS83561.1	AP3/DEF
Eupteleaceae			
Euptelea pleiosperma			
	EupPI	ADC79697.1	PI/GLO
	EupAP3-1	AEG25813.1	AP3/DEF
	EupAP3-2	ADC79696.1	AP3/DEF
Papaveraceae			
Eschscholzia californica	EscaGLO	ABN55897.1	PI/GLO
	EscaDEF1	ABN55895.1	AP3/DEF
	EscaDEF2	ABN55896.1	AP3/DEF
	EscaDEF3	CCC54332.1	AP3/DEF
Ranunculaceae			
Nigella damascena	NdPI1	ALM95515.1	AP3/DEF
	NdPI2	ALM95516.1	PI/GLO
	NdAP3-1	ALM95512.1	AP3/DEF
	NdAP3-2	ALM95513.1	AP3/DEF
	NdAP3-3	ALM95514.1	AP3/DEF
Paeoniaceae			
Paeonia lactiflora	PLPI1	AQM56615.1	PI/GLO
	PLPI2	AQM56626.1	PI/GLO
	PLAP3-1	AQM56588.1	AP3/DEF
	PLAP3-2	AQM56599.1	AP3/DEF
Vitaceae			
Vitis vinifera	VvPI	ABK59993.1	PI/GLO
	VvAP3	ABN71371.1	AP3/DEF
	VvTM6	ABI98021.1	AP3/DEF
Fabaceae			
Medicago truncatula	MtPI	ACJ36228.1	PI/GLO
	MtNGL9	ACJ36229.1	PI/GLO
	MtNMH7	AEW43601.1	AP3/DEF
	MtTM6	AEW43602.1	AP3/DEF
Lotus japonicus	LjPIa	AAX13299.1	PI/GLO
	LjPIb	AAX13300.1	PI/GLO
	LjAP3	AAX13301.1	AP3/DEF
Rosaceae			
Kerria japonica	KjPI	AXR86369.1	PI/GLO
	KjAP3	AXR86368.1	AP3/DEF
Eriobotrya japonica	EjPI	QGR27129.1	PI/GLO

	EjAP3	QGR27127.1	AP3/DEF
Malus domestica	MdPI	CAC28021.1	PI/GLO
	MdMADS13	CAC80856.1	AP3/DEF
	MdTM6	BAC11907.1	AP3/DEF
Violaceae			
Viola philippica	VpPI	APQ46145.1	PI/GLO
	VpTM6-1	APQ46143.1	AP3/DEF
	VpTM6-2	APQ46144.1	AP3/DEF
Brassicaceae			
Arabidopsis thaliana	AtPI	BAA06465.1	PI/GLO
	AtAP3	P35632.1	AP3/DEF
Polygonaceae			
Fagopyrum esculentum	FaesPI_1	OM032616.1	PI/GLO
	FaesPI_2	OM032617.1	PI/GLO
	FaesAP3_1	AFO83616.1	AP3/DEF
	FaesAP3_2	QLJ83023.1	AP3/DEF
Primulaceae			
Primula vulgaris	PvGLO1	AQT19636.1	PI/GLO
	PvGLO2	AQT19638.1	PI/GLO
	PvDEF1	ABD78317.1	AP3/DEF
	PvTM6	AZZ09244.1	AP3/DEF
Sapotaceae			
Argania spinosa	AsPI	UDH08096.1	PI/GLO
Actinidiaceae			
Actinidia chinensis	AcPI	ADU15475.1	AP3/DEF
	AcAP3-1	ADU15473.1	AP3/DEF
	AcAP3-2	ADU15474.1	AP3/DEF
Gentianaceae			
Gentiana scabra	GsPI1	BAS04475.1	PI/GLO
	GsPI2	BAS04476.1	PI/GLO
	GsPI3	BAS04478.1	PI/GLO
	GsAP3a	BAS04474.1	AP3/DEF
	GsAP3b	BAS04479.1	AP3/DEF
	GsTM6	BAS04472.1	AP3/DEF
Solanaceae			
Physalis pubescens	PFGLO1	AGN54427.1	PI/GLO
	PFGLO2	AGN54426.1	PI/GLO
	PFDEF	AGN54423.1	AP3/DEF
	PFTM6	AGN54424.1	AP3/DEF
Lycopersicon esculentum	TPI	ABG73411.1	PI/GLO
	TAP3	ABG73412.1	AP3/DEF

	TDR6	NP_001311309.1	AP3/DEF
Oleaceae			
Osmanthus fragrans	OfGLO1	QCS90129.1	PI/GL
Scrophulariaceae			
Antirrhinum majus	GLO	CAA48725.1	PI/GLO
	DEF	BAI68389.1	AP3/DEF
Bignoniaceae			
Catalpa bungei	CabuPI	AJY60427.1	PI/GLO
Asteraceae			
Gerbera hybrid	GGLO1	CAA08804.1	PI/GLO
	GDEF1	CAA08802.1	AP3/DEF
	GDEF2	CAA08803.1	AP3/DEF
	GDEF3	ACV53813.1	AP3/DEF

> *pFaesPI_1*

-2186 C**AGAAA**TCCT TAACTCCTTC CTTCCCAGCC T**TACT**AA**CAA ATG**TAGCTGA GATCTCCTT**C**

POLLEN1LELAT52 CACTFTPPCA1 MYCCONSENSUSAT

-2126 **CAAT**TGTAAG TTTGCTTGCT TCCGTTTTTC TTTTGAAAGT CATTTAAAAT **TGAC**ACTAGA

CCAATBOX1 WRKY71OS

-2066 AGTTTAACTA GTTCAAAAGT CCAAGGAAAA TTTGGACCCC TA**CACT**TTT**G TGA**GAGATTA

CACTFTPPCA1 GTGANTG10

-2006 TTATTGTCCA TCTACAAGCT CAAAA**CAAT**A TAA**CATATG**T TTAGTACA**AA CAAA**ACACCA

CAATBOX1 MYCCONSENSUSAT AACAAA motif

-1946 CAAAATGAAA AATAAAAAAT GTATAAGATT AGGTA**CACT**G GAAGCATTCT AAAAATCAGA

CACTFTPPCA1

-1886 TTGGGTA**CAC T**GCAAGCATT TA**TACT**GGTA TAAGAATAGA GAAGTTTCTC AAACTATCGA

CACTFTPPCA1 CACTFTPPCA1

-1826 TGGATCATAC ATACAAAACT GCAG**CAAT**AA ATTCTTAACT T**CAAT**CTCAA ACTATCGATG

CAATBOX1 CAATBOX1

-1766 AATATAA**AAC CTAAC**AGT**TT ATTT**GAAGTT TAGCAGCAGT TCCACAAATA TCAGACCACA

MYBPLANT TATABOX5

-1706 CAAG**AACAAA** GATTAAATGG AGCTTAAATG GACA**CAATTG** CAAGAGTATG AAGAAGTTTC

AACAAA motif MYCCONSENSUSAT

-1646 AGCAACAGTC TAACTT**TAAC AAA**GAT**TATA AAT**GGACA**CA ATTG**CAACCC ATAAATTACA

MYBGAHV TATABOX2 MYCCONSENSUSAT

-1586 GAAGT**CAAT**A GTCACAAAAT TACAACTCTG AAGAGAAGAG TAAATTACAT CAGTTTGGAA

CAATBOX1

-1526 ATAAAATGTA ATAAAGGAAG CAACCCGAAG CA**CAATTG**CT CAA**CAAT**CAA GCAAACGCAG

MYCCONSENSUSAT CAATBOX1

-1466 CAACTACAAC CTTATGGTAA GAATAATCAT A**CCAAT**TTCA GTAGATAGAA CATGTATACA

CCAATBOX1

-1406 GAC**TGAC**CCT TGGCTAGTAT CAAATTCAAA ATGGAAGCAT TATATATTAA ACATGAACTC

WRKY71OS
-1346 AGATTCGAAC AGAGCAGT**CA AT**AACT**CACT** GCAAATTATA AACTCAGATT CGAAAAGCAA
CAATBOX1 CACTFTPPCA1
-1286 CCCGAAGCAA CTACAAGTTT CCACATTCAT TTTA**GTGA**TA ACAAGTTTCC ACATT**CATTT**
GTGANTG10 CArG-box
-1226 **TTTAG**TGATA ACAACATGTA TACAGACTAA CCCTTGGCTA GTATCAAATT CGAAATGGAA
-1166 GCATTATATT AAACATGAAC TCAGATTCGA ACAGAGCAGT **CAAT**AACT**CA CT**GCAAATTA
CAATBOX1 CACTFTPPCA1
-1106 TAAACTCAGA TTCGTTTAAT CTGAATAGAT GCAGGAAGAC TCAGATTA**TA CT**ACAAAATC
CACTFTPPCA1
-1046 TAATAACTCA CTGCAGCCAA GTTA**TGAC**TC AGATTCGAAA AGCAAAATAG ATGCAGGAAC
WRKY71OS
-986 TCAAATTATA AACTCAGATT TGAG**CAAT**CA CCACCATCAG ATTCGAATTG GAAAGAACAG
CAATBOX1
-926 TCACCACCAT CAGATTCGAA TTCGAAAAGC AAAACAGATT CG**AACAAA**AC AGATTCGAAC
AACAAA motif
-866 AGAGCAGTCA CCACCGTCAG ATTCGAACAG ATTCGAATAG ATGCAGGAAC TCGAATAGAT
-806 GCAGGAACTT **CAAATG**CATT G**CAAT**CA**CAA ACAC**GGTTTA AGGGTTTTGA TGAGAATAT**T**
MYCCONSENSUSAT CAATBOX1 CANBNNAPA
-746 **ACT**GTAATGC AG**CAAT**CCGA AACGTACCTC AAACTTGTCG **CCTTTT**CTCG TCCTCTTGCC
CACTFTPPCA1 CAATBOX1 PYRIMIDINEBOXOSRAMY1A
-686 GCCGTCAGTT TCTCTTTG**CC GTTG**CCGTCG CAGGGTTGCT CCAGATCGGG GAAGAAGATA
MYBCORE
-626 GCAAGGTTTG ATGAGAGAGG GTGTTAATTA TATACC**CGGT TA**ATGCGTAA TTCTTGTTAA
MYBCORE
-566 TAGATGA**GGA TA**TTTTGGGT ATTTTGCACG G**AGAAA**CCCT AGTGTAACCC CAAAACTCTT
MYBST1 POLLEN1LELAT52
-506 GCT**CAGATG**G GAGTTTCACA AA**CAAT**CGG**A GAAA**CACTAA TCACCACTGT TTCTCTCCGT
MYCCONSENSUSAT CAATBOX1 POLLEN1LELAT52
-446 GC**CAAACAC**T CGTAAAAACG AGATA**CACT**A CAAAA**CAAT**A AAAAA**CAAT**A GTAGTATCCG
CANBNNAPA CACTFTPPCA1 CAATBOX1 CAATBOX1
-386 TGTGC**CAAAC AC**AACCTTAG CTTTTTGTAG AG**AGAAA**CAT GTACGAATAG GTAGCTAAGT
CANBNNAPA POLLEN1LELAT52
-326 TAACCT**AGAA A**TGTGTCTAG AGGAAAACTC AGACTTGTAT GAATCTCATA TACAGCTAGC
POLLEN1LELAT52
-266 TAGCTATGGA **GGATA**GAAGG GACCAA**CGGT TA**ACAAGGTG **CAAT**AGCATT TTCTCATACA
MYBST1 MYBCORE CAATBOX1
-206 AAG**CAAT**GTT TTTTG**GTGA**A AATTCAGCAA GGTAAAATTG AAAAGACA**AT TTCAAA**ACAA
CAATBOX1 GTGANTG10 ERELEE4
-146 AGA**AGAAA**AA AAACCCATTT AA**AACCAACC** TTTTTG**CACT** CTCTT**AGAAA** GTCTAT**TGTC**
POLLEN1LELAT52 MYBPLANT CACTFTPPCA1 POLLEN1LELAT52 ARFAT
-86 **TC**TT**AAACCC TA**TATAGTAA GCATCT**CACT** GTACTCATCT TCTTCCCC**CA CT**C**CACT**TTA
UP2ATMSD CACTFTPPCA1 CACTFTPPCA1 CACTFTPPCA1

+1

−26 ATTTTCACAT CCACC**CACT**T CTTTTC**A**TTT GTCTTCTTCA CCTTCTTCCA TTATAATTTA

CACTFTPPCA1

+35 CAACACAG**AG AAA**AACTAGG G**TTTGTT**TTT GGAG**ATG**

POLLEN1LELAT52 TTTGTT motif

Supplementary Figure S1. *pFaesPI_1* promoter sequence.

The transcription start site (+1) is in bold and boxed. The start codon ATG is in bold and boxed. Putative cis-acting regulatory elements are in bold and underlined.

> ***pFaesPI_2***

−2057 CTCGAAGGTA TGAAGGAATA **TGAC**ACGAAG GTTGCTGCTG CTTGCTACCA TGGCCTCCAG

WRKY71OS

−1997 AT**CCAATGTT** GATGTCGGCT GCAGAGGAGG AACGAGGCTA GACT**CAACTG** GAGGGGTTCG

LEAFYATAG MYCCONSENSUSAT

−1937 AGTGTTTCCT GAAGAGATCG ACG**CAGATG**G CCTGGGGACG GGGCTGTA**GT GA**CTTGCTAG

MYCCONSENSUSAT GTGANTG10

−1877 CGCTATCCAT GGCTGAGGAC GAGGCTGGA**G TGA**CCTGCTA GCGCCATCCA TGGCTGAGGA

GTGANTG10

−1817 CGAGGCTGGA **GTGA**CCTGCT AGCGCCATCC ATGGCTGAGA ACAGGGCTGG A**GTGA**ACTGC

GTGANTG10 GTGANTG10

−1757 TAGCGCCATC CATGGCAGAG TGCAGAAGCC GGCGTCCAAG GAGGAGGTCC AGTCCAGGTA

−1697 GACGGCTGCT ATT**GTGA**GCC CTCACAC**GTG A**AGAACGCTT GTTGCTGCTT GAGTAGAGGG

GTGANTG10 GTGANTG10

−1637 CTGGAAGTT**C CGAC**TGTGCA GATCAGGAGG GGGAAAAGAG ATAGGTTGTA GGTAGAAGGT

LTRECOREATCOR15

−1577 TGTGTAGTAT ACCATGGATT AAATGTTTGA GTAAGTTCAG AAG**GTGA**CGA TGAATAAG**CG**

GTGANTG10

−1517 **GTTA**CGAAAA GGGGAGGTGT GTGGATTCTG TACGGGTTTT AGAAGGAAAG AGAGGTATTT

MYBCORE

−1457 GATAGGATCT ATTTATTCCC TGAATATACG GTAGGCTGGA ATGCTTCATT GCTGGCTGGT

−1397 AGAACCTGGC AGCT**TGGGCT** GAAGCATAAG GGTGCTAGAT CT**TGGGCT**TT GTCA**TGGGCT**

SITEIIATCYTC SITEIIATCYTC SITEIIATCYTC

−1337 AGTCATGAGC TTGTCAAGGA TTTGTCA**TGG GCT**TGT**CAAA TG**GCCTTATT GCCTTGTCAA

SITEIIATCYTC MYCCONSENSUSAT

−1277 AAGGTCCATC AAATTCTACA TTTTCAAAAG TGTGTTATAA GTAGTATT**TT ATTT**GATCAA

TATABOX5

−1217 AACTCCACAT AAAATCATAA AAGTT**TGTAA AG**ATCATTTT ATGTGTTTAA TTCATCACAT

-300CORE

−1157 AAAATACCAA GAT**TTATTT**T GACTTGTAAA TGAACGGAA**A GAAA**AAAAAT TTC**CGGTTA**T

TATABOX5 POLLEN1LELAT52 MYBCORE

-1097 TACATTTGGC CTACGAGAGG GCCTCCAAAA GGCCGCCAAC ATTGGTATTC TATCCATATT
MYCCONSENSUSAT

-1037 GAAGGTGACA ACCTTACGCT AATCAATGTA GTTAACCAAC TATGGAAGGC ACCATGGGGA
GTGANTG10 CAATBOX1 MYB1AT

-977 TGGATATGAT CATCTCGGAC ATCTCCACCT TGATATCCAG TTTTGAGCGG TTCTACGCTC
MYBST1

-917 AACATATATT TAGAGAAATC AACGTCGTAG CAGACAGACT TGCATTGCAT GATTTTGACC
POLLEN1LELAT52 WRKY71OS

-857 CGGCAATAAA GACGAAGTGC TACCGGATGA CTTTGGATGC TGTGAGAATG ACGGTTTTTA
CAATBOX1 WRKY71OS GTGANTG10 WRKY71OS

-797 GGAACTAACC TTACCACTTC GCTTACCCTT GTATCAAAAA GAAAAAGAAA AAAAAAAAAA
CACTFTPPCA1 POLLEN1LELAT52 POLLEN1LELAT52

-737 TTTGGTTGCA GCCAAACAAG CCCTAGGGAT TGGAAGTATT CTTCATTTTT GACAGCAGCT
WRKY71OS

-677 TTTGACCATG TGGATGTGGT GGGAGACTGC AGTAATTTCC TGAACCCATT TTCTTCCTTT
WRKY71OS

-617 GACCCGACCC ATTTCCAAAA CCATGTAAAA AAAAAAGAAA AAAAATAAAC GTCGCCCACA
LTRECOREATCOR15 MYB1AT POLLEN1LELAT52 ABREOSRAB21

-557 TATATGTCTA TCCTTTGCTG ATCGTACAAG AAAACAATAA ACTTGAGTTG ATATATTGAT
POLLEN1LELAT52 CAATBOX1

-497 TTGATGGTTA GGATCATGTG TATATTGCTG TGTCTCCTTC AGATTTCCCT CCAAACCCTA
MYCCONSENSUSAT TELOBOXATEEF1AA1

-437 ATTGACATTT TGACATGACC CATTTTATCT CTCAGTCAGC TACACTACAA ACTTCTTTTC
WRKY71OS WRKY71OS WRKY71OS CACTFTPPCA1

-377 ATTTCACATC TCCTTATTAG ACTATAATTC TCTATTTATC ACTCTCATAA CAAAATCTTC
CACTFTPPCA1 AACAAA motif

-317 ATCATCTTAA AATACTCGAA TACTAAAATT AAAAACTCAG TAATTAACTC AATATATCAA
CACTFTPPCA1 CAATBOX1

-257 CTCAAGTTTT TTGTAGAGAG AAACATGTAC GAATAGGTAG CTAGGTTAAC CTAGAAATAT
POLLEN1LELAT52 POLLEN1LELAT52

-197 GTCTAGAGGA AAACTCAGCA AGGTAAAATT GAAAAGACAA TTTCAAAATA AAGAAGAAGA
CAATBOX1 CArG-box

-137 AAAACCCATT TAAAACCAAC CTTTTTGCAC TCTCTTAGAA AGTCTATTGT CTCTTAAACC
POLLEN1LELAT52 MYBPLANT CACTFTPPCA1 POLLEN1LELAT52

-77 CTATATAGTA AGCATCTCAA TGTACTCATC TTCTTCCCCC ACTCCACTTT AATTTTCACA
UP2ATMSD CAATBOX1 CACTFTPPCA1 CACTFTPPCA1 CACTFTPPCA1

+1

-17 TCCACCCACT TCTTTTTATT TGTCTTCTTC TCGTTCTTCC ATTACAATTT ACAACACAGA
CACTFTPPCA1 CAATBOX1

+44 GAAAAACTAG GGTTTGTTTT TGGAGATG
POLLEN1LELAT52 TTTGTT motif

Supplementary Figure S2. *pFaesPI_2* promoter sequence.

The transcription start site (+1) is in bold and boxed. The start codon ATG is in bold and boxed. Putative cis-acting regulatory elements are in bold and underlined.

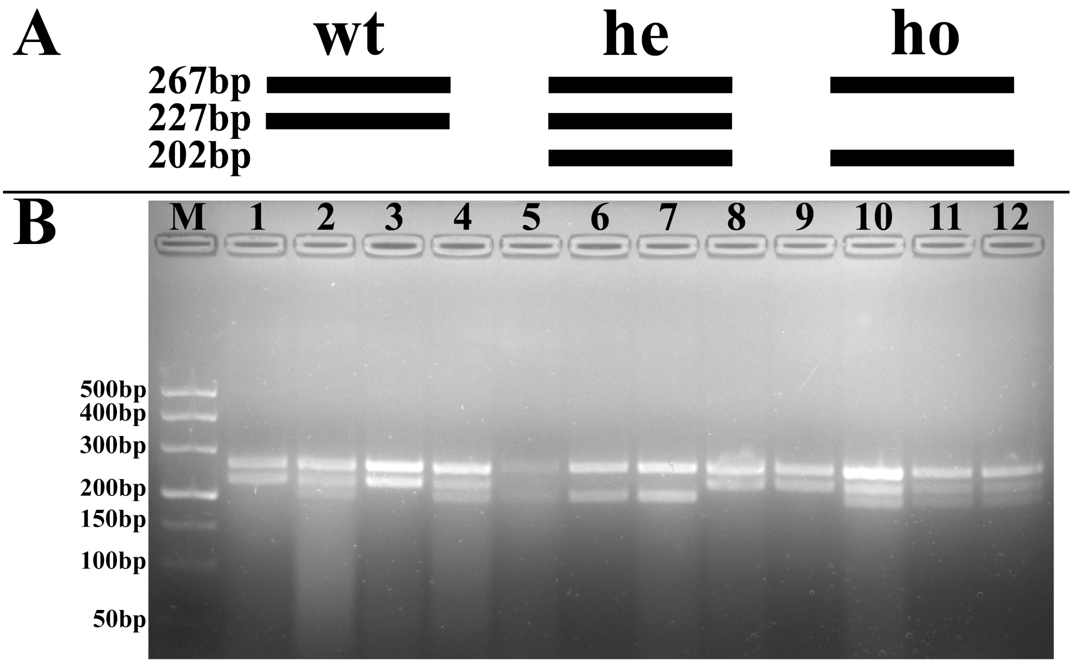

Supplementary Figure S3. Genotyping of wildtype, heterozygous and homozygous *pi-1* mutant *A. thaliana* by dCAPS.

The amplicons from transgenic lines in wild-type background *Arabidopsis* were cleaved by *BspHI* to produce 267 bp and 227 bp fragment, and the amplicon from heterozygous *PI/pi-1* background plants were cleaved by *BspHI* to produce 267 bp, 227 bp and 207 bp; the amplicon from transgenic lines.
